# Solid-State Supercapacitors with Enhanced Performance Using Al^3+^-Doped Li^+^ Ion Perovskite Electrolyte Integrated with Carbon Aerogel Electrode

**DOI:** 10.1021/acsomega.5c05602

**Published:** 2025-08-21

**Authors:** Bhargab Sharma, Hardeep, Kamaldeep Bisht, Ashish Singh, Rashmi Singh, Anshuman Dalvi

**Affiliations:** † Department of Physics, 29794 Birla Institute of Technology and Science, Pilani, Pilani Campus, Vidya Vihar, Pilani, Rajasthan 333031, India; ‡ Photonic Materials Technology Section, 62392 Raja Ramanna Centre for Advanced Technology, Indore 452013, India

## Abstract

We report the performance of solid-state ceramic supercapacitors (SSCs) based on a novel composite electrolyte comprising aluminum-doped lithium lanthanum titanate perovskite, Li_0.36_La_0.56_Ti_0.995_Al_0.005_O_3_ (Al^3+^-doped LLTO), and the ionic liquid 1-ethyl-3-methylimidazolium tetrafluoroborate (EMIM BF_4_). Rietveld refinement of X-ray diffraction data confirms the preservation of the tetragonal perovskite phase after Al^3+^ substitution, indicating structural stability of the host lattice. X-ray photoelectron spectroscopy (XPS) and Raman spectroscopy further corroborate the successful incorporation of Al^3+^ without forming secondary phases. The addition of ∼6 wt % EMIM BF_4_ into Al-LLTO matrix significantly enhances the room-temperature ionic conductivity to ∼10^–3^ Ω^–1^ cm^–1^, nearly 3 orders of magnitude greater than that of pristine LLTO, resulting into improved long-range electrical transport. Further, novel SSCs have been fabricated by sandwiching the composite electrolyte between high surface area freeze-dried carbon aerogel (FD-CA) coated copper electrodes and assembled using a low-cost hot-roll lamination approach. The devices at 35 °C exhibited a high specific capacitance of ∼370 F g^–1^ at 1 mA/2 V, excellent cycling stability with ∼87% capacitance retention over 15,000 cycles at 2 V and 2 mA (1 A g^–1^), and stable Coulombic efficiency of ∼99%. These symmetric SSCs demonstrate ideal electric double-layer capacitive behavior for operating potential ≤ 2 V. These results highlight the potential of Al^3^-doped LLTO/EMIM BF_4_ composite electrolytes in combination with FD-CA-based electrodes for the development of safe, efficient, stable and scalable solid-state supercapacitors.

## Graphical abstract

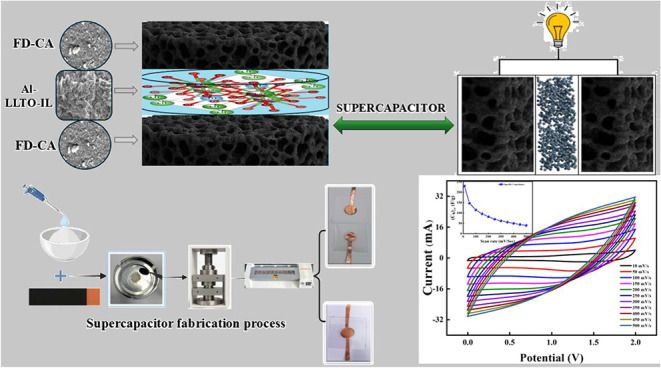

## 1 Introduction

The growing demand for a wide range of power applications in society has encouraged researchers to delve into high-energy and power-density energy storage devices.^1^ High energy density devices, such as batteries, excel due to the chemical reactions at the electrode–electrolyte interface, delivering consistent long-term energy output. In contrast, high power density devices like supercapacitors are engineered for rapid energy delivery, making them ideal for quick bursts of power. This dual approach ensures that the evolving energy needs of our society are effectively met.^2,3^ Efforts have shifted toward hybrid energy storage devices to synergistically combine high energy density batteries with high power density supercapacitors, paving the way for safer and more efficient energy storage systems. Among the energy storage systems, liquid and gel electrolyte-based batteries and supercapacitors based on solid-state electrolytes are at the forefront of technology, showcasing their vital role in immediate applications.^4^ Meanwhile, solid-state batteries and supercapacitors have also shown promise to revolutionize the next generation of energy storage solutions. This is due to their superior safety features, broader temperature tolerance, wider electrochemical stability window, longer shelf life, and enhanced performance compared to conventional systems currently used in commercial technology.^5^ Therefore, the ongoing research on energy storage systems also emphasizes the development of advanced solid electrolytes (fast ionic conductors)^6^ and engineering their interface with the electrodes.

Fast ionic conductors (solid electrolytes) have been effectively used in solid-state batteries, but their potential applications in supercapacitors have not been thoroughly explored.^7^ So far, the focus in supercapacitor development has been on liquid or gel-based devices, primarily concentrating on creating a wide spectrum of high surface area materials.^2^ However, the liquid electrolytes used in these supercapacitors lead to several limitations, including safety risks such as flammability, volatility, leakage, and corrosion. Additionally, they have a restricted electrochemical stability window and poor thermal stability.^8^

Fast ionic ceramic materials have demonstrated an increasingly vital potential in energy storage devices due to appreciably very high chemical stability, wide voltage stability windows, and mechanical strength. In context to solid-state batteries, ceramic electrolytes e.g., garnet-type Li_7_La_3_Zr_2_O_12_ (LLZO), NASICON-type Li_1+*x*_Al_*x*_Ti_2–*x*_(PO_4_)_3_, and perovskite-type Li_3*x*_La_2/3–*x*_TiO_3_ (LLTO) conduct Li^+^ ions at room temperature (σ ≥ 10^–4^ Ω^–1^ cm^–1^) without the safety risks of liquid electrolytes. However, each class has its pros and cons: NASICON frameworks can offer three-dimensional ion pathways but need very precise aliovalent doping to remain stable, whereas garnets resist lithium dendrite penetration but suffer high grain-boundary impedance, and perovskites are chemically tunable and comparatively less reactive than the other two but are intrinsically brittle. By compositing these ceramics with polymers or conductive fillers, researchers have so far aimed to optimize the ionic conductivity, controlling the electrolyte membrane thickness, mechanical stability, and reproducibility.^7,9,10^

Solid-state supercapacitors based on liquid-free composite solid polymer electrolyte membranes have been developed recently.^11–13^ On the other hand, solid-state ceramic supercapacitors (ASSC) typically sandwich a thin sheet of fast ionic ceramic electrolyte film between porous electrodes, enabling rapid ion transport and excellent and long cycling stability. Prior to use in the devices, the interface of the ceramic electrolyte with the electrodes requires a tailoring of impedance that is achieved either by adding ion conductive polymer or ionic liquid (IL) in a small amount.^14–16^

Among the promising materials for energy storage devices, perovskite-structured Li^+^ ion solid electrolytes have attracted considerable attention due to their exceptional electrochemical properties, including high ionic conductivity, tunable band gaps, and excellent stability in comparison to the NASICON or garnet-structured Li^+^ ion conducting ceramics.^17^ These perovskite materials also hold great potential for use as electrolytes in supercapacitors in addition to batteries.^18–21^ In fact, for the first time, our group developed ionic liquid (IL) dispersed Li_0.34_La_0.51_TiO_2.94_ (LLTO) perovskite composite electrolytes for use in activated carbon-based SSCs, which showed high specific capacitance 312 Fg^1–^ at 2 V/2 mA with stable Coulombic efficiency (η∼99%) and 60% capacitance retention in 10000 galvanostatic charge–discharge cycles.^22^ During this investigation, we also realized that pristine LLTO demonstrates a low ionic conductivity at room temperature, which possibly limits the SSC performance. However, the low conductivity of LLTO is compensated by the IL presence in the matrix. The IL not only improves interfacial contacts but also overall electrical transport. Since the IL amount cannot be increased beyond ∼5–6 wt % in the matrix, the SSC performance can only be improved if LLTO conducting composition is altered by some substitution. For example, the substitution of elements, e.g., Al^3+^, also increases the overall conductivity of the pristine LLTO electrolyte remarkably as reported by various groups. For instance, as per an investigation, a small amount of 0.5 wt % Al substitution for Ti results in a 23 wt % increase in conductivity.^23^ Moreover, the grain boundary impedance of LLTO can be reduced significantly by incorporating secondary phases into the LLTO precursor powders, such as Li_7_La_3_Zr_2_O_12_,^24^ Al_2_O_3_,^25,26^ and SiO_2_. These added materials primarily serve to improve conduction across the grain boundaries, which hinder the movement of lithium ions into a rapid ion migration pathway known as the space charge layer at the interface of the two phases.

In addition to electrolytes, electrode materials play a pivotal role in the performance of supercapacitors and need a thorough investigation. Carbon-based porous materials have emerged as highly suitable for electrodes due to their excellent chemical stability, electrical conductivity, cost-effectiveness, and environmentally benign nature.^27–29^ Activated carbon (AC) is amorphous, randomly porous, and predominantly microporous. Since the mesoporous surface area plays an important role in the cycling stability of the device, AC has low cycling stability and low capacitive retention. Among these, nanoporous carbon aerogels derived from organic precursors such as resorcinol-formaldehyde or phenolic resins stand out for their tunable porosity and surface area properties, which are governed by their synthesis and process parameters. Carbon aerogels (CA) exhibit several distinctive properties of high surface area typically ranging from 500 to 1500 m^2^/g, good electrical conductivity, chemically robust and mechanical flexibility. The thermal and chemical stability of CA further contributes to their long-term durability, which is an essential attribute for the stable cycling performance of supercapacitors. Such properties make CA ideal candidates for use as electrode materials in electric double-layer capacitors (EDLCs). Interestingly, the three-dimensional open porous network of carbon aerogels provides an extensive mesoporous network that facilitates efficient charge storage, thereby enhancing the capacitance of supercapacitor devices. An important step in carbon aerogel synthesis is the availability of mesopores, which leads to better ion transport pathways for enhanced supercapacitor performance. Various methods have been used for enhancing mesoporous e.g., Critical point drying (CPD) and freeze-drying (FD) techniques provide superior properties with minimal shrinkage.^30^ The CPD process operates under high-pressure and high-temperature conditions, posing safety concerns and limiting scalability. In contrast, FD is performed at low pressure and temperature, offering a safer and more scalable alternative suitable for bulk production. To enhance electrochemical performance further, effective integration of carbon aerogels with various electrode materials such as graphene, carbon nanotubes, and polymers has been observed in recent years.^31^

It was therefore envisaged that the combination of CA with fast ionic materials such as perovskite-type electrolytes may have the potential for a synergistic approach where the high ionic mobility and electrochemical activity of perovskites are coupled with the structural integrity and conductivity of carbon aerogels. The resulting composite electrodes are expected not only to deliver enhanced energy and power densities but also to superior charge/discharge rates and remarkable cycling stability, with minimal electrode degradation over extended use. The perovskite-carbon aerogel composite supercapacitor is particularly appealing for those applications that demand long-term stability and reliability.

In the present study, with an aim to enhance the ionic conductivity, first, Al^3+^-substituted lithium lanthanum titanate (Al-LLTO) was synthesized.^24^ As a second step, a minimal amount (∼6 wt %) of the ionic liquid 1-ethyl-3-methylimidazolium tetrafluoroborate (EMIM BF_4_) was introduced in Al-LLTO to improve interfacial contact between the electrode and electrolyte. For the electrode material, freeze-dried carbon aerogel (FD-CA) was prepared via a sol–gel polymerization process using resorcinol and formaldehyde as precursors. Various structural, thermal, and electrical characterizations were used for the electrode and electrolyte. A solid-state supercapacitor was subsequently fabricated using the synthesized electrode and electrolyte materials. The assembled device was subjected to comprehensive electrochemical characterization in order to test the various performance parameters.

## 2 Experimental Section

### 2.1 Materials

Lanthanum­(III) nitrate hexahydrate (La­(NO_3_)_3_·6H_2_O, Thermo scientific 99.9%), Lithium nitrate (LiNO_3_, Thermo scientific 99%), Aluminum nitrate (Al­(NO_3_)_3_, Alfa Aesar 99%), Titanium­(IV) n-butoxide (Alfa Aesar, 99%), Ethylene glycol monomethyl ether (Thermo scientific, 99%), 1-ethyl-3-methylimidazolium tetrafluoroborate (EMIM BF4, Thermoscientific, 99%), Resorcinol (Thermo scientific, 99%), Formaldehyde (Thermoscientific, 99%), Sodium Carbonate (Thermo scientific, 99%) were procured from various distributor as mention and used without further purification.

### 2.2 Preparation of Al-LLTO Perovskite Electrolyte

The perovskite-type Li_0.36_La_0.56_Ti_0.995_Al_0.005_O_3_, referred to as Al-LLTO hereafter, has been synthesized by using the sol–gel route.^22^ A stoichiometric amount of lithium nitrate (LiNO_3_), lanthanum­(III) nitrate (La­(NO_3_)_3_·6H_2_O), and aluminum nitrate (Al­(NO_3_)_3_) are dissolved in ethylene glycol monomethyl ether, with thorough stirring at 40 °C for 1 h. To this solution, titanium­(IV) butoxide and acetylacetone are added in a 1:1 molar ratio. The final solution is then stirred at 60 °C for approximately 6 h, resulting in a brownish gel-like mixture. This mixture is heated to 200 °C for 2 h, yielding a black and white ash-like compound, which is then crushed and further calcined at 900 °C for 6 h. The resulting white powder is then calcined at 1200 °C for 5 h, leading to the formation of Al-LLTO white powder. (detailed steps, Figure 1a). The Aluminum-doped LLTO ionic liquid (Al-LLTO-IL) composite was synthesized by uniformly mixing Al-LLTO powder with ionic liquid (IL) using a planetary ball mill (Fritsch-P6) for 1 h in an agate pot. The mass to ball ratio of the sample was 5:1. The ionic liquid used for the composite preparation was EMIM BF_4_, in weights ranging from 1 to 6 wt %. The samples are abbreviated as Al-LLTO-*x*IL, where *x* represents the weight percentage of IL. It is important to note that beyond 6 wt %, the IL becomes excess and separates as a liquid. Optimal measurements were conducted for the composite containing approximately 6 wt % IL in the LLTO matrix. From here on, the Al-LLTO-6 EMIM BF4 composite will be referred to as Al-LLTO-IL.

**Figure 1:**
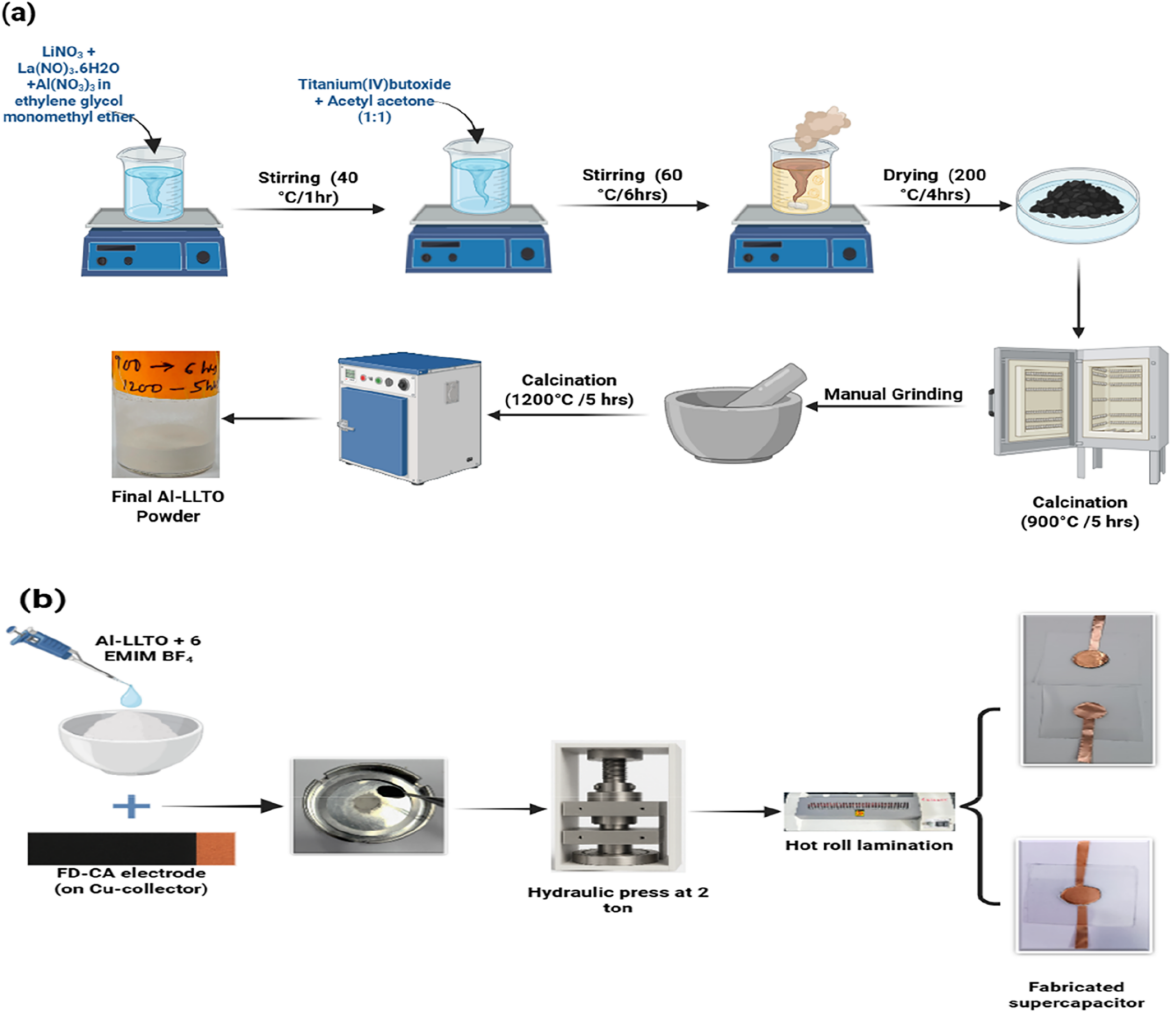
(a) Schematic diagram for the preparation of the Al-LLTO composite electrolyte. (b) Solid-state supercapacitor fabrication process. (“Photograph courtesy of Bhargab Sharma. Copyright 2024”).

### 2.3 Preparation of Freeze-Dried Carbon Aerogel (FD-CA)

Carbon aerogel material was prepared by sol–gel polymerization of resorcinol and formaldehyde in an aqueous medium.^30^ Initially, Resorcinol (R) was dissolved in deionized water (W) with an R/W molar ratio of 0.12. Sodium carbonate (Na_2_CO_3_) was added as a catalytic agent (C) for gelation at R/C molar ratio of 400. Subsequently, formaldehyde (F) was mixed at an R/F molar ratio of 0.5, and the resulting sol was placed in an oven for gelation at 65 °C. The aquagel obtained was cured at 80 °C for 65 h. After curing, the aquagel underwent freeze-drying (FD) in which the gel was frozen at – 20 °C for 4 to 6 h, and sublimation was carried out under a vacuum of 10^–2^ to 10^–3^ bar without the formation of any liquid interface. Finally, the dried gel was pyrolyzed at 800 °C for 2 h in an inert atmosphere to produce the freeze-dried carbon aerogel, referred to hereafter as FD-CA. The carbon aerogel was activated using CO_2_ gas at 920 °C for 3 h to further enhance its surface properties. The electrodes were prepared with FD-CA as the active material, PVDF-HFP as the binder and Acetylene Black as the electronic conductor in a ratio of (75:15:10) in *N*-Methyl-2-pyrrolidone (NMP) solvent, which is further coated in a copper strip.

### 2.4 Characterization

X-ray diffraction (XRD) patterns of Al-LLTO, Al-LLTO-IL composite, and freeze-dried carbon aerogel (FD-CA) were recorded using a Rigaku Miniflex II diffractometer with Cu Kα radiation (λ = 1.54 Å) to identify crystalline phases and structural changes. Surface morphology and microstructural features of Al-LLTO, Al-LLTO-IL, and FD-CA were investigated using field emission scanning electron microscopy (FESEM) (FEI-Apreo-Lovac). Thermogravimetric analysis (TGA) was performed using a DTA-60 Series (Shimadzu) in a nitrogen atmosphere from 30 to 600 °C at a heating rate of 10 °C min^–1^ to assess thermal stability and composition. X-ray photoelectron spectroscopy (XPS) (Thermo Scientific K-α) was employed to analyze the oxidation states, chemical environments, and surface elemental composition of Al-LLTO, Al-LLTO-IL, and FD-CA. The Brunauer–Emmett–Teller (BET) surface area of the FD-CA was measured using a BELSORP-MINI X analyzer. Ionic/electrical conductivity measurements were carried out using a HIOKI IM3570 impedance analyzer over a frequency range of 4 Hz to 5 MHz and a temperature range of 40–220 °C under steady-state conditions to evaluate electrochemical performance.

### 2.5 Supercapacitor Fabrication

The Al-LLTO-IL composites were further tested under supercapacitor conditions using FD-CA electrodes. The electrode slurry preparation, coating, and drying process to obtain the final electrodes is shown in Figure 1b. The mass loading per electrode was kept precisely to 1 mg cm^–2^, and electrodes of 14 mm in diameter were cut into circular shapes for SSC fabrication. Al-LLTO-6IL composite electrolyte was placed between FD-CA electrodes and put under pressure of up to ∼2 tons cm^–2^ in a hydraulic press. The sandwiched configuration was transferred to laminated supercapacitor geometry, as shown in Figure 1b. To characterize these SSCs, different techniques such as electrochemical impedance spectroscopy (EIS), cyclic voltammetry (CV), and galvanostatic charge–discharge (GCD) cycling were carried out using an electrochemical workstation Autolab 204. Long Cycling of the SSC was carried out using the NEWARE battery testing system.

## 3 Results and Discussion

### 3.1 X-ray Diffraction Analysis

Rietveld refinement of the Al-doped LLTO is shown in Figure 2a. The refinement exhibits a good fit with a chi-squared (χ^2^) value of 1.92, which indicates reliable model accuracy. The X-ray diffraction (XRD) pattern confirms the formation of a tetragonal perovskite structure belonging to the space group *P*4/*mmm*.^32^ Since the dopant concentration of aluminum is very low, the overall perovskite framework is preserved, and no significant structural distortion is detected in the XRD pattern. Figure 2c illustrates the perovskite-type crystal structure of Al-LLTO generated using VESTA software based on the output of the Rietveld refinement. The structure adopts a tetragonal symmetry consistent with the space group *P*4/*mmm*.^33^ The refined parameters are found to be *a* = *b* = 3.877 Å, *c* = 7.757 Å and unit cell volume, *V* = 116.596 Å^3^. These values confirm the formation of a tetragonal perovskite phase, in agreement with the X-ray diffraction data. The structure shows no evidence of impurity phases or secondary phase precipitation, suggesting that Al^3+^ ions have been successfully substituted at Ti^4+^ sites within the lattice, which suggests successful doping. This structural stability further corroborates the homogeneous doping of Al^3+^, maintaining the crystallographic structure of the perovskite. Figure 2b shows the XRD pattern of the Al-LLTO and Al-LLTO-IL composites. The XRD pattern indicates that there are no noticeable changes after adding ionic liquid (IL) to the Al-LLTO. This clearly suggests that the ionic liquid does not form any new crystalline compound. This observation is consistent with the results we reported earlier for the LLTO-IL composite.^22^ The X-ray diffraction (XRD) pattern of the FD-CA sample is shown in Figure 2d. Broad diffraction peaks were observed at 19.5̊ and 42̊, which corresponds to the (002) and (101) planes, respectively. The (002) peak is associated with the interplanar spacing of the graphitic layers, indicating the degree of graphitization and the presence of stacked carbon sheets. The (101) plane corresponds to the in-plane ordering of carbon atoms within the graphene-like layers. The broad nature of these peaks suggests an amorphous carbon structure with limited crystallinity and short-range order, which is a typical characteristic of disordered carbon materials.^30^

**Figure 2:**
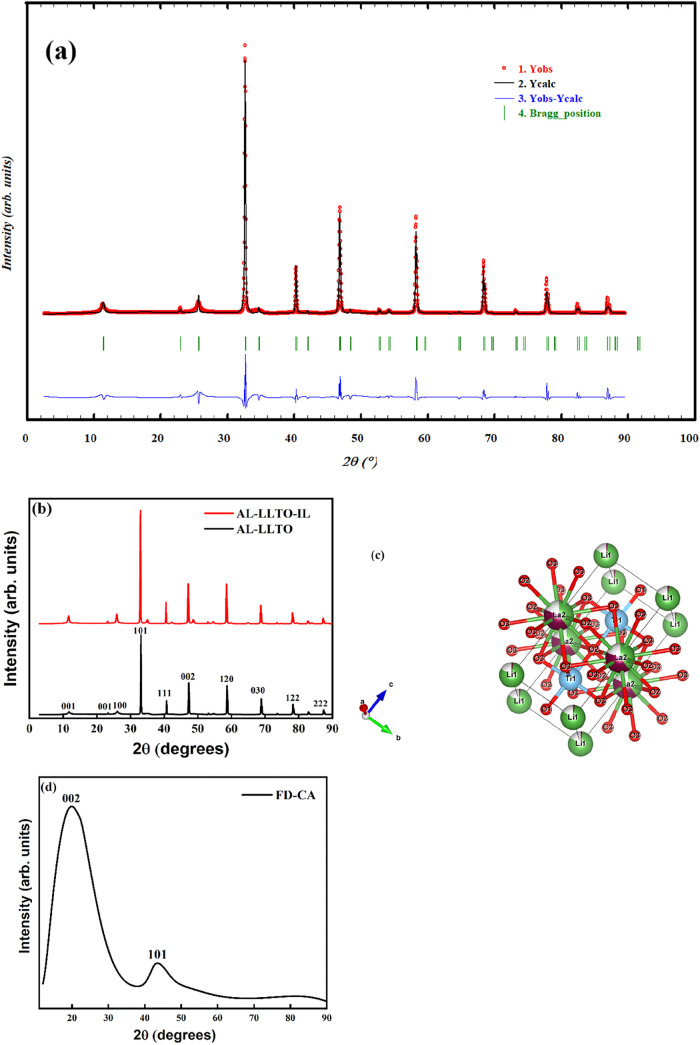
(a) Rietveld refinement of Al-LLTO, (b) XRD patterns of Al-LLTO and Al-LLTO-IL, (c) Al-LLTO structure obtained after refinement, (d) XRD the pattern of FD-CA.

### 3.2 FESEM Analysis

The field emission scanning electron microscopy (FESEM) images of the Al–LLTO and Al–LLTO–IL composite, as shown in Figure 3a,b, reveal that the grains are uniformly distributed with no signs of aggregation.^34,35^ Furthermore, there is no significant change in the grain size with the incorporation of the ionic liquid (IL), which suggests that Al–LLTO maintains its structural integrity at the interface with the IL. Figure 3d presents the elemental color mapping of the Al–LLTO–IL composite, clearly demonstrating the presence of the ionic liquid distributed among the Al–LLTO grains. The distribution of ionic liquid is very homogeneous and is at the grain–grain interface of the Al-LLTO. Elemental mapping also indicates that the IL does not segregate but exhibits a uniform dispersion within the composite matrix. The corresponding energy-dispersive X-ray spectroscopy (EDS) spectrum, shown in Figure 3e, further confirms the elemental composition and indicates that impurity levels are negligible. Additionally, the FESEM images do not show any evidence of grain swelling, suggesting that the ionic liquid does not penetrate the Al–LLTO grains but remains localized at the interface. Figure 3c presents the FESEM image of FD-CA, revealing a highly porous network with a nanostructured carbon framework. The morphology exhibits a sponge-like or web-like architecture, characterized by interconnected pores that are advantageous for facilitating efficient and long-range ionic transport.

**Figure 3:**
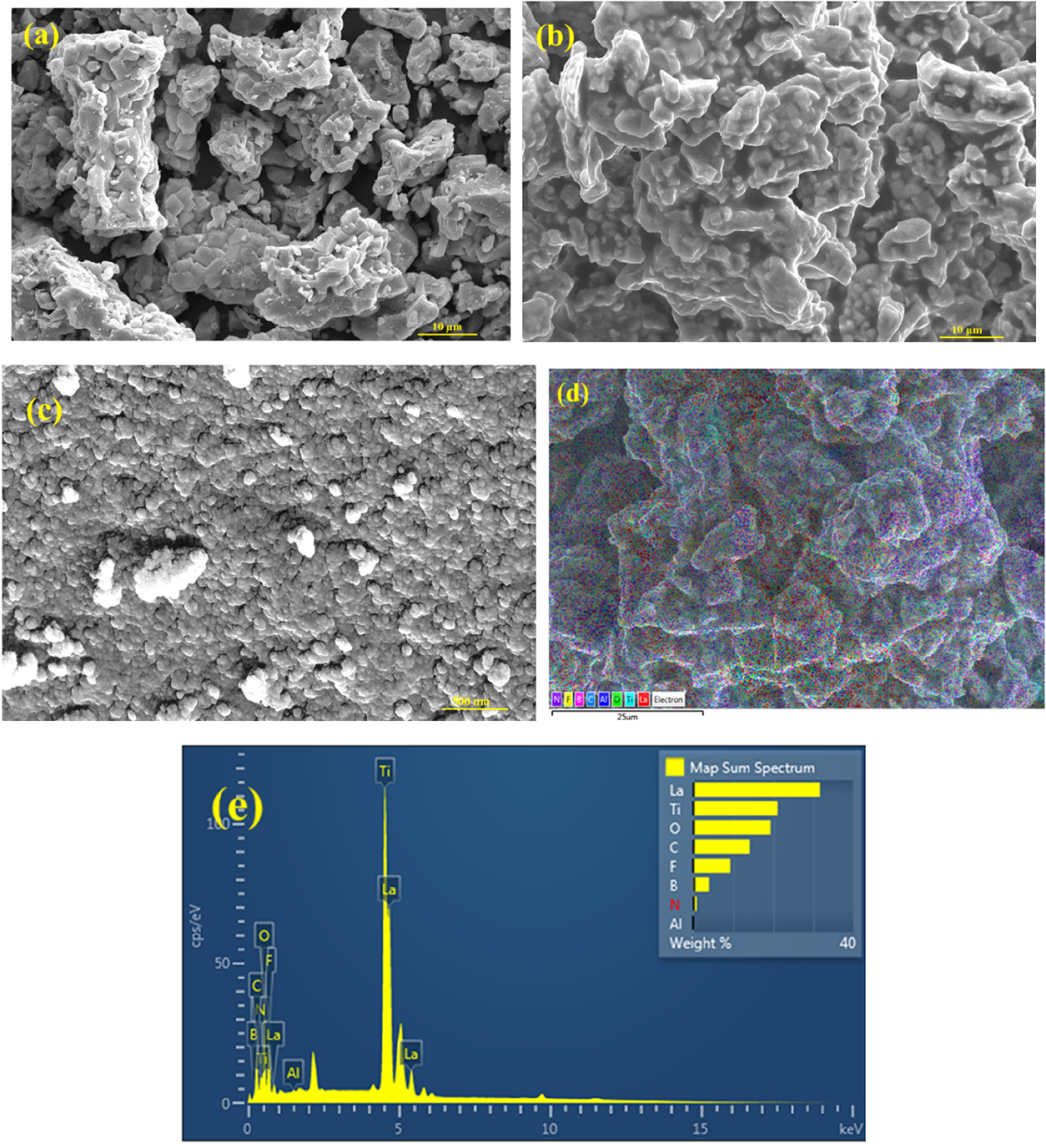
FESEM images of (a) Al-LLTO, (b) Al-LLTO-IL, (c) FD-CA, (d) and (e) Al-LLTO-IL EDS elemental mapping (main Figure in Supporting Figure S3).

### 3.3 TGA Analysis

The thermogravimetric analysis (TGA) profiles of pristine Al-LLTO and the Al-LLTO-IL composite are compared in Figure 4a. The pristine Al-LLTO shows no sudden weight loss as the temperature increases. Instead, a gradual weight reduction from 100% to approximately 98% is observed as the temperature rises from room temperature to 600 °C. This slight decrease in weight is attributed to the gradual removal of adsorbed water and residual organic compounds, which decompose gradually over this temperature range. In contrast, the Al-LLTO-IL composite exhibits a significant weight loss starting around ∼350 °C, corresponding to the decomposition of the ionic liquid (IL). Up to ∼350 °C weight loss is rather gradual readily suggesting the gradual nature of this weight loss suggests that the IL, along with some water, is physically adsorbed onto the surface of the LLTO rather than being bonded chemically. This readily indicates that, within the composite, the IL is held primarily through weak physical interactions of varying strengths rather than through strong covalent or ionic bonds. Figure 4b illustrates the Thermogravimetric Analysis (TGA) of FD-CA. A noticeable weight loss is observed around 100 °C, which can be attributed to removing physically adsorbed or bound water from the sample. Beyond this point, the FD-CA exhibits thermal stability over a wide temperature range, indicative of its high thermal resilience, structural robustness, and integrity.

**Figure 4:**
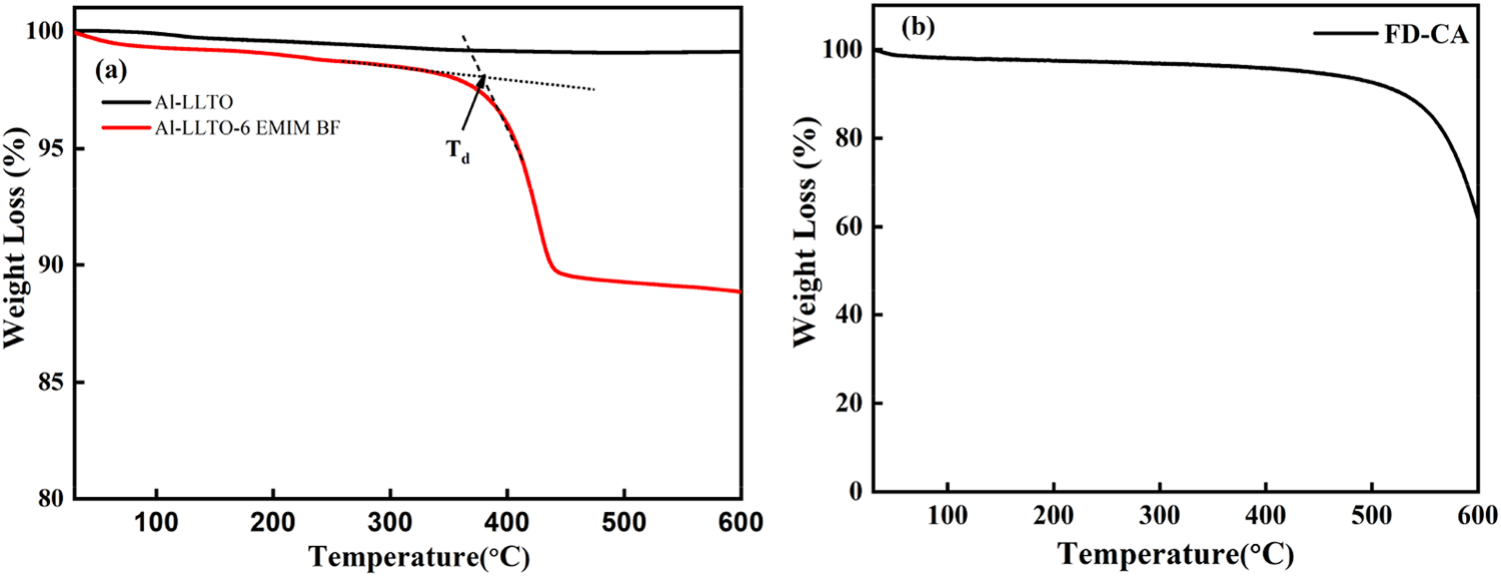
TGA profile of (a) Al-LLTO and Al-LLTO-IL, (b) FD-CA.

### 3.4 Raman Spectroscopy

The Raman spectra of Al-LLTO and the Al-LLTO-IL composite are presented in Figure 5a. Six characteristic bands are observed at approximately 140, 238, 315, 452, 526, and 597 cm^–1^, which are consistent with the tetragonal structure of Al-LLTO.^36^ The bands at ∼142 and 315 cm^–1^ are attributed to Ti vibrations along the in-plane and *c*-axis directions, respectively. The bands at ∼238 and 526 cm^–1^ are assigned to oxygen-related vibrational modes. Additionally, an Al-associated peak appears near 452 cm^–1^. Compared to pure LLTO, peak shifting occurs after the addition of Aluminum due to the changes in the Ti–O vibrational mode and the mass difference between Ti and Al. Moreover, such structural modifications are expected to influence the electrical transport of the Al-LLTO.^37^ Raman spectroscopy of the FD-CA sample was performed to see the degree of graphitization, as shown in Figure 5b. Two broad peaks characterize the spectrum centered at approximately 1340 and 1590 cm^–1^. According to Tinstra and Koenig, graphitic materials typically exhibit a G-band near 1575 cm^–1^ and an additional D-band around 1355 cm^–1^, depending on the structural order. The relative intensities of these bands are sensitive to the degree of graphitization and the presence of structural defects. The peak at ∼1355 cm^–1^ corresponds to the A_1_g vibrational mode, commonly referred to as the D-band, which arises from disorder and the breakdown of translational symmetry. The peak near 1575 cm^–1^ is attributed to the E_2_g vibrational mode, the G-band, associated with the in-plane stretching of sp^2^-bonded carbon atoms in the graphene layer. In the case of FD-CA, the observed peaks at ∼1340  and 1590 cm^–1^ are therefore assigned to the D-band and G-band, respectively, indicating the presence of disordered graphitic domains.

**Figure 5:**
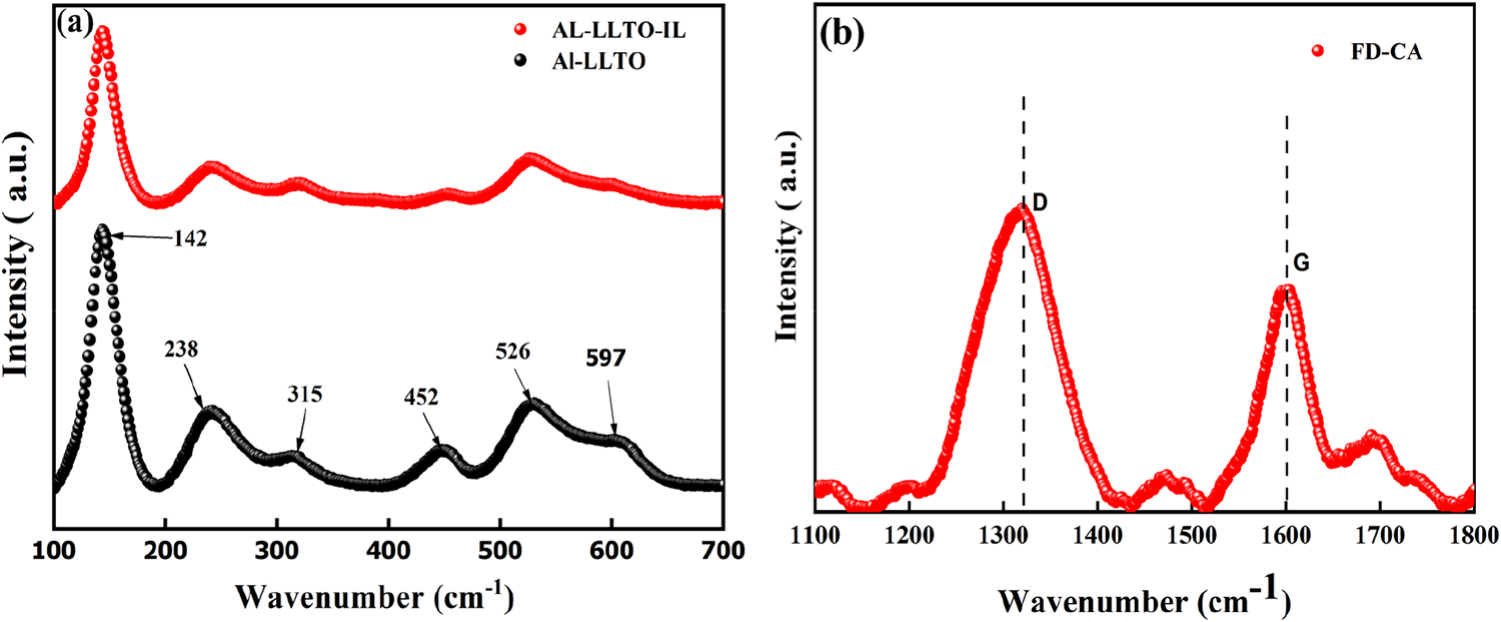
Raman spectra of (a) Al-LLTO and Al-LLTO-IL, (b) FD-CA.

Figure S1 presents the BET isotherm with relevant details, which confirms the high mesoporous surface area of the FD-CA.

From the above discussion, it may be suggested that the incorporation of Al^3+^ in the LLTO matrix is successful, and the tetragonal structure is retained. FD-CA also exhibits G and D band which confirms the presence of graphitic domains.

### 3.5 XPS Analysis

Figure 6a–c shows the X-ray photoelectron spectroscopy (XPS) survey spectra of Al-LLTO, Al-LLTO-IL, and FD-CA. All characteristic elemental peaks are observed at their respective binding energies, with no unidentified or impurity peaks detected, indicating the high chemical purity of the samples. Peak deconvolution was performed using CasaXPS software (version 2.3.19), employing the U 2 Tougaard background correction model. For the Al-LLTO sample, the deconvoluted La 3d spectrum reveals distinct spin–orbit doublets at binding energies of approximately 834 eV (La 3d_5/2_) and 855 eV (La 3d_3/2_). Satellite peaks ∼4 eV above the main peaks confirm the presence of the La^3+^ oxidation state. No noticeable effect of Al doping on the La environment was observed. In the case of the Al-LLTO-IL composite, the La^3+^ oxidation state remains unchanged, indicating that interaction with the EMIM^+^ ionic liquid does not induce any valence change in La. The Ti 2p spectrum for Al-LLTO shows spin–orbit doublets at approximately 458 eV (Ti 2p_3/2_) and 464 eV (Ti 2p_1/2_), with a splitting of ∼6 eV, which is characteristic of the Ti^4+^ oxidation state originating from TiO_6_ octahedra.^38,39^ Similarly, in Al-LLTO-IL, no change in the Ti oxidation state is observed. A single symmetric peak in the Al 2p region confirms the absence of metallic Al or secondary aluminum oxide phases, suggesting successful substitutional doping of Al^3+^ at Ti sites. The oxidation state of aluminum is confirmed as Al^3+^. The substitution of Ti^4+^ (ionic radius ∼ 0.605 Å) with smaller Al^3+^ ions (ionic radius ∼0.535 Å) reduces Ti–O bond length, thereby enhancing bond strength and possibly improving lattice stability. The O 1s peak centered at ∼532.35 eV is higher than that of typical lattice oxygen (∼529.5–530 eV), indicating the presence of surface species such as hydroxyl groups or molecular oxygen associated with organic moieties. Additionally, the presence of C 1s peak at ∼286.08 eV suggests either the existence of oxygenated carbon species or nitrogen-containing functional groups, likely originating from the EMIM^+^ cation. These observations confirm the intact and stable presence of EMIM BF_4_ on the composite surface and the successful doping of Al^3+^ without reduction.^40^

**Figure 6:**
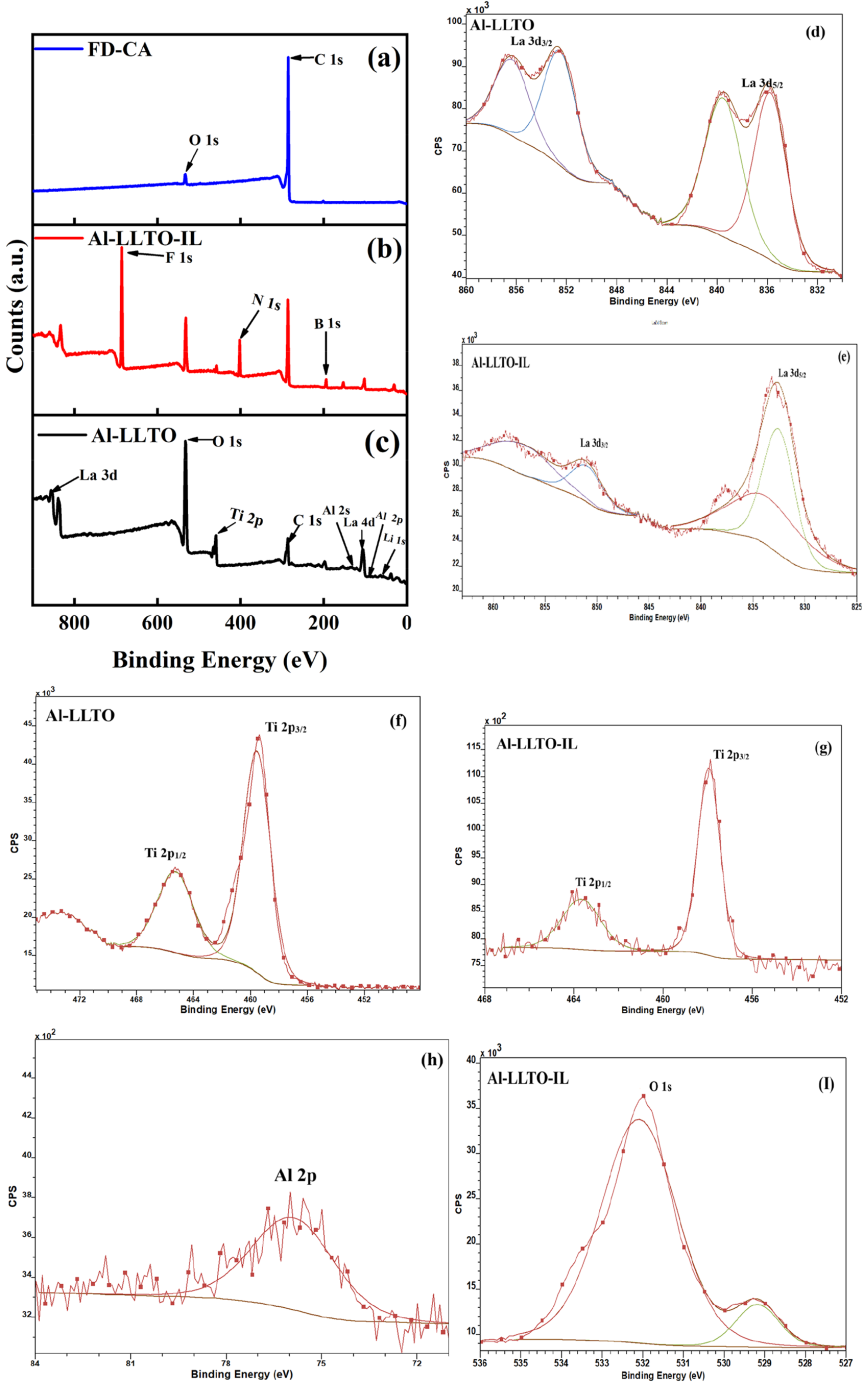
XPS survey spectra of (a) FD-CA, (b) Al-LLTO-IL, (c) Al-LLTO. Deconvolution peaks of (d) La 3d for Al-LLTO, (e) La 3d for Al-LLTO-IL, (f) Ti 2p for Al-LLTO, (g) Ti 2p for Al-LLTO-IL, (h) Al 2p, (i) O 1s.

The XPS spectrum of the FD-CA aerogel, therefore, indicates the absence of detectable impurities. High carbon content affirms effective carbonization, while minor oxygen peaks are attributed to surface functional groups or residual oxygen from incomplete carbonization. A summary of the key characteristics of the Al-LLTO-IL composite is presented in Table 1.

**Table 1:** Summary of the Key Characteristics of the Al-LLTO-IL Composition

element	core Level	BE (eV)	chemical state	role
C	1s	286.08	—	C–O/C–N bonds (EMIM^+^), adventitious carbon
N	1s	402.05	N^+^	imidazolium nitrogen in EMIM^+^
F	1s	686.09	F^–^	fluorine in BF_4_^–^ anion
B	1s	194.08	B^3+^	boron in tetrahedral BF_4_^–^
Ti	2p_3/2_	458.10	Ti^4+^	octahedral TiO_6_ in LLTO
La	3d_5/2_	850.84	La^3+^	A-site cation in the perovskite lattice
Al	2p	73.65	Al^3+^	substitutional doping at Ti^4+^ site
Al	2s	116.97	Al^3+^	confirmatory signal
Li	1s	52.08	Li^+^	interstitial/mobile Li^+^ ion
O	1s	532.35	O^2–^/OH^–^	lattice oxygen and surface hydroxyls

### 3.6 Electrical Transport

First, the steady-state electrical conductivity of the composites was measured and plotted as a function of frequency. The DC conductivity was extracted from the σ-ω plateau as shown by the interception of the dotted line, Figure 7a point for both the Al-LLTO and Al-LLTO–IL composites. Figure 7b shows the temperature dependence of the dc conductivity for the pristine LLTO and Al-LLTO. For the Al-LLTO sample, the temperature dependence of conductivity exhibits Arrhenius behavior with an activation energy of 0.31 eV. The value matches with other reports, which is less than the activation energy of LLTO with 0.63 eV.^22^

**Figure 7:**
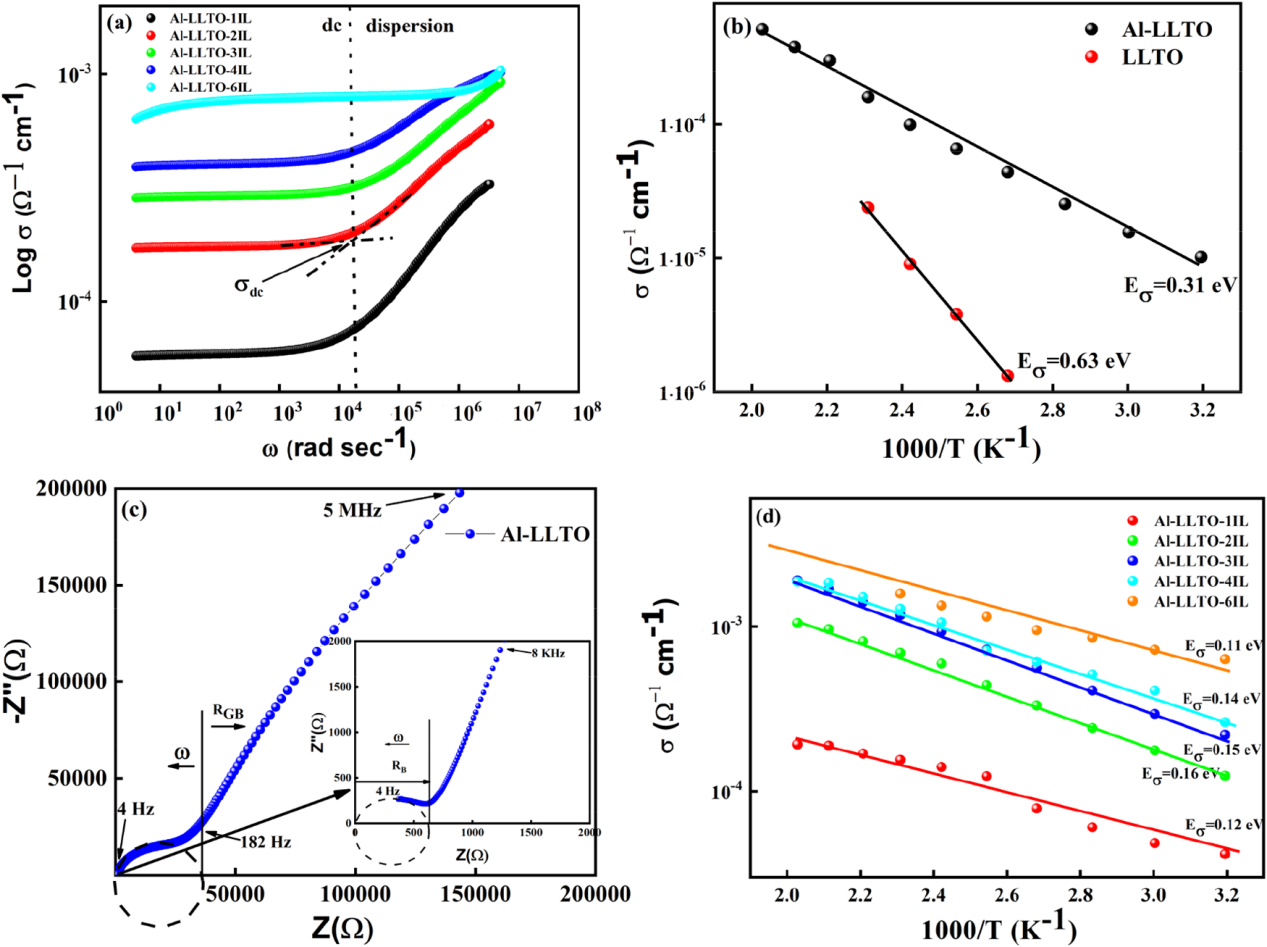
(a) Frequency dependence of the electrical conductivity of Al-LLTO and Al-LLTO-xIL, (b) Temperature dependence of the total conductivity of LLTO and Al-LLTO, (c) Nyquist plot of Al-LLTO at 40 °C, inset depicts high-frequency region on an extended scale, (d) temperature dependence of total conductivity for all the composites having Al-LLTO and varied amounts of EMIM BF_4_.

As evident from the data, the incorporation of Al^3+^ significantly enhances the ionic conductivity by approximately 2 orders of magnitude, reaching ∼10^–5^ Ω^–1^ cm^–1^ at 40 °C. In the tetragonal perovskite structure of LLTO, Li^+^ ions occupy two types of sites, designated as B_1_ and B_2_.^32^ The occupancy of these sites is influenced by the substitution of trivalent Al^3+^ for tetravalent Ti^4+^. This substitution creates a charge imbalance, which is compensated by incorporating additional mobile Li^+^ ions that preferentially occupy the B_2_ sites. This substitution enhances mobile charge carriers and disrupts the ordering of Li^+^ and La^3+^, thereby facilitating better pathways for ion transport and increasing the overall ionic conductivity. A similar effect is observed in NASICON-structured Li_1.5_Al_0.5_ Ti_1.5_(PO_4_)_3_ where replacing Ti^4+^ with Al^3+^ results into an increase in mobile Li^+^ ion content and improves ionic conductivity through the same charge-compensation mechanism.^41^

Although doping with Al^3+^ improves the ionic conductivity, the observed values are still insufficient for practical device applications. This limitation is attributed to the high grain boundary impedance (GBI). Mobile Li^+^ ions encounter substantial resistance during long-range diffusion across grain boundaries. While the bulk conductivity in such polycrystalline systems can be intrinsically high, the GBI substantially diminishes the overall ionic conductivity, as reported for NASICON- and garnet-type electrolytes.

The Nyquist plot Figure 7c clearly shows a fairly low bulk (in-grain) and significant grain boundary impedance in Al-LLTO, making it inadequate for supercapacitor fabrication. To tailor the GBI, the ionic liquid EMIM BF_4_ was introduced into the Al-LLTO matrix at various content wt % as shown in Figure 7d. As discussed, the pristine Al-LLTO exhibits an activation energy E_σ_ ∼ 0.31 eV, which is notably lower than that of undoped LLTO. Upon the addition of 1 wt % IL, the activation energy decreases remarkably, accompanied by a significant increase in conductivity. The optimal conductivity (∼10^–4^ Ω^–1^ cm^–1^) for any device application was achieved at 5 wt % IL. However, beyond a threshold amount (6 wt %), phase segregation occurred, and the IL began to leach out from the pellet. These observations suggest that the high GBI in pristine LLTO poses a considerable energy barrier to ionic transport, which is substantially mitigated by the incorporation of IL. The electrical transport behavior aligns with our prior IL–NASICON^42^ and IL–LALZO^17^ composites studies.

It may be inferred from the above discussion that

(i) The ionic liquid (IL) occupies the spaces between the grains and the electrode–electrolyte interface. It may also and may diffuse to the grain boundaries due to concentration gradient and temperature effects. However, structural investigations using X-ray diffraction (XRD) and X-ray photoelectron spectroscopy (XPS) clearly show that the IL does not penetrate into the grains, or unit cells. Additionally, it does not react with the ceramic at the interface. Thermogravimetric analysis (TGA) also suggests that the IL is physisorbed onto the AL-LLTO interface.
(ii) Total conductivity in the composites may include contributions from ionic liquid (IL) ions, along with lithium ions (Li^+^) supplied by Al-LLTO from the ceramic. In a variety of previous investigations have been reported where conductivity enhancement was achieved by dispersing little amount of IL (≤10 wt %) into ceramics such as in IL-Li_2_S–P_2_S_5_ glass,^43^ IL-Li_2_SO_4_–LiPO_3_,^44^ IL-LATP,^41^ and IL-LALZO.^17^ According to these reports, since the amount of IL is minimal and the size of IL ions is relatively large (5–15 Å), long-range diffusive motion of IL-derived ions is unlikely.
(iii) In a separate study on an ionic liquid (EMIM BF_4_) mixed with a lithium ion sulfide glassy system (Li_2_S–P_2_S_5_)^45^ It was found that the ionic liquid is capable of dissolving the mechanically stronger glassy phase when its content is ≥ 70 mol %. In the current composite, the amount of IL is small (≤10 wt %), but its interaction with the ceramic (Al-LLTO) interface may liberate mobile Li^+^ ions and facilitate their movement along the interface or through the grains when an electric field is applied.
(iv) Further, the impedance spectroscopy measurements conducted on IL-LATP and LALZO composites, particularly when the IL is present in small amounts, have revealed a single conductivity relaxation that corresponds solely to Li^+^ ions. An interesting study by Hayashi et al.^46^ demonstrated that the mobility of Li^+^ ions is enhanced in the Li_2_S–P_2_S_5_ glass matrix when it is added with IL (EMIM BF_4_) in a small amount. Further, the Li^+^ ion transport number in the composite is approximately 0.74.^47^ Therefore, while the system predominantly conducts Li^+^ ions, the IL ions do not contribute to the total conductivity.

The change in activation energy of ionic transport is rather gradual from 0.31 eV (0 wt % IL) to 0.11 eV for (6 wt % IL), which also suggests that Li^+^ ions are the majority charge carriers contributing to the electrical transport. Since supercapacitors do not require a single ion motion, such a composite with mixed mobile ions can be effectively applied for electrolytic usage.

### 3.7 Electrochemical Characterization

At the outset, Figure 8a presents the Linear Sweep Voltammetry (LSV) of the Al-LLTO-IL composite electrolyte to determine its electrochemical stability window. It is observed that the electrolyte (with graphite electrode) remains stable up to ∼2.8 V, beyond which a sharp rise in current indicates decomposition. In comparison, the stability of electrochemical working voltage window of Al-LLTO (inset of Figure 8a) is limited to ∼2.4 V. These results clearly demonstrate that incorporation of the ionic liquid extends the stability window to ∼2.8 V against graphite electrode. To ensure safe and reliable operation of the device, a maximum operating voltage of 2 V was chosen, well within the stability range of the electrolyte.

**Figure 8:**
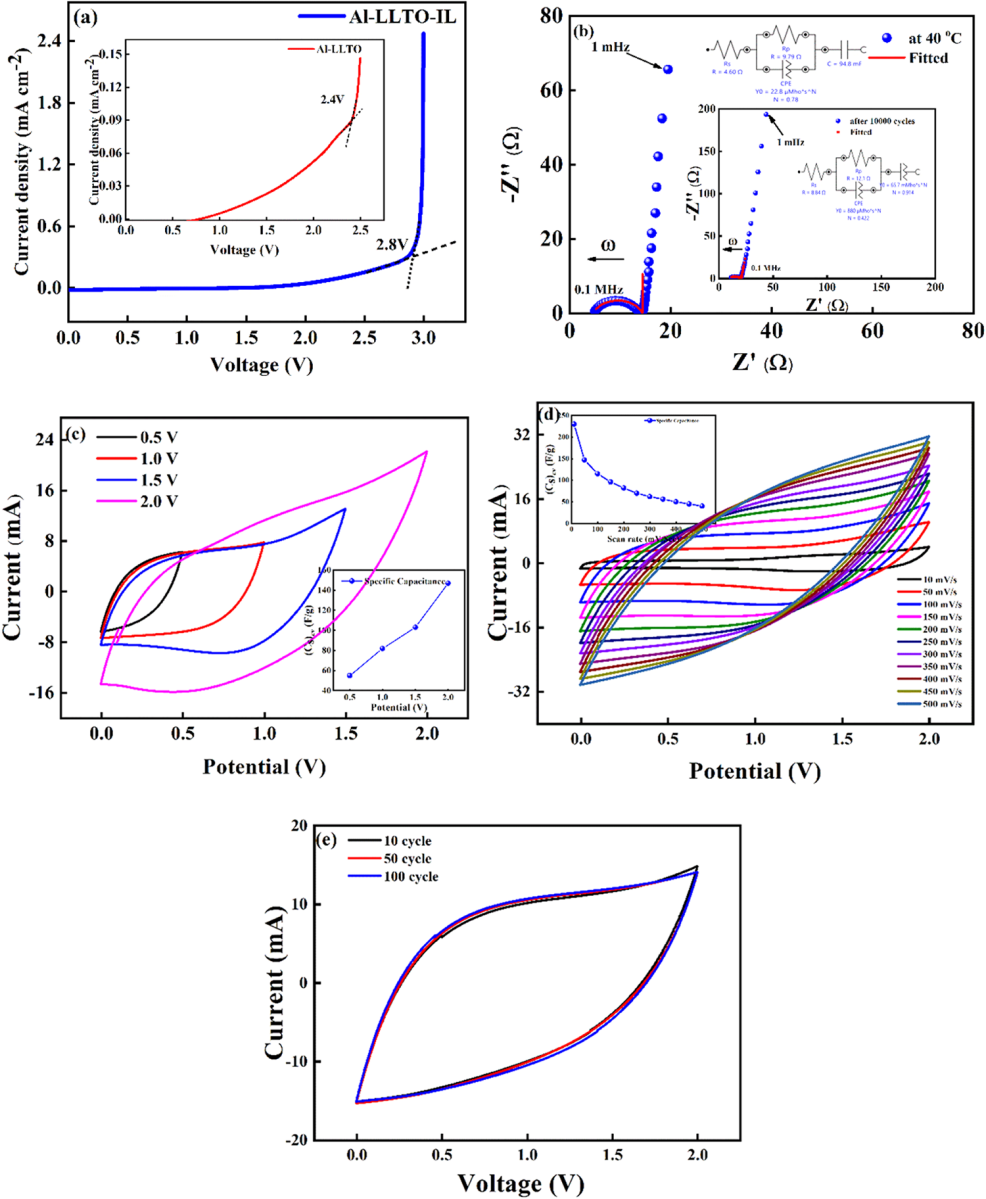
Electrochemical workstation analysis of Al-LLTO-6IL SSC with FD-CA as a electrode. (a) Linear Sweep Voltammetry (LSV) against graphite electrode of Al-LLTO-IL; Inset: LSV of Al-LLTO­(b) Electrochemical impedance spectroscopy (EIS) of SSC with fitted circuit; Inset: EIS of SSC after 10,000 GCD cycles, (c) Cyclic voltammetry (CV) of the SSC at different voltages; Inset: Specific capacitance from CV, (d) CV at different scan rate; Inset: Specific capacitance from CV. (e) 100 cycles of CV at 2 V and 100 mV Sec^–1^.

The SSCs are further characterized using electrochemical impedance spectroscopy (EIS) and cyclic voltammetry (CV). In the EIS technique, Nyquist plots present imaginary and real components.

Typically, at high frequencies (MHz region), the semicircular portion of the plot corresponds to the charge transfer process, with the diameter of the semicircle representing the charge transfer resistance (*R*_ct_) at the electrode–electrolyte interface. In contrast, the vertical straight-line slope in the low-frequency region reflects the mobile ion diffusion and adsorption, reflecting the capacitive characteristics of the active material at electrodes.^48,49^ Figure 8b shows the EIS plot in a wide frequency range (1 mHz–1 MHz). From the equivalent circuit fitting, the series resistance (*R*_s_) is determined to be 4.60 Ω, indicating low bulk resistance affected by the Al-LLTO-IL composite electrolyte, contributing a little to the overall resistance of the device. The polarization resistance (*R*_p_, the resistive component of the electrochemical interface) is found to be as low as 9.79 Ω, suggesting efficient charge movement across the electrode–electrolyte interface and, thus, favorable electrochemical performance. The inset of Figure 8b shows the EIS spectrum of the SSC after 10,000 galvanostatic charge–discharge (GCD) cycles. Remarkably, the bulk resistance of the electrolyte changes only to a value of 8.84 Ω, confirming that the interface maintains smooth ionic transport even after prolonged cycling. Further, the *R*_p_ after 10,000 cycles is measured to be 12.1 Ω, indicating that the charge transfer process at the interface remains efficient. This sustained performance can be attributed to numerous active sites on the FD-CA framework, which provides continuous ion transport pathways and contributes to the excellent cycling stability of the SSC.

Figure 8c presents the cyclic voltammetry (CV) profiles recorded at 100 mV/s and 40 °C with progressively increasing voltage up to 2 V, aimed at determining the optimal electrochemical window. The CV curves remain featureless and nearly rectangular and featureless up to 2 V, indicating ideal capacitive behavior and electrochemical stability. At lower voltages, through the CV adopts a leaf-like shape, possibly due to reduced ionic mobility and electrode kinetics. Figure 8e further illustrates the CV stability over 100 cycles at 2 V and 100 mV/s, confirming excellent electrochemical durability with no noticeable degradation. The CV curves were also used to assess the capacitance using the relation $\left(C\right)cv=\frac{\int i\left(V\right)\mathrm{dV}}{\left(2\;\times \;scan\;rate\;\times \;\Delta V\right)}$ Where ∫*i* (*V*)*dV* is the area under the CV curve, Δ*V* is the potential window (V_2_–V_1_) used for the scan, and the scan rate is the rate at which the potential (voltage) applied to the working electrode is swept over a specified range. The specific capacitance per electrode is then calculated by $Cs=\frac{2\left(C\right)\mathrm{cv}}{m}$ where *m* is the average mass of the active material on one electrode. For the 10th cycle (2 V, 100 mV/s), the specific capacitance from the CV curve at 40 °C is around ∼115 F/g. The Onset of Figure 8c gives the *C*_s_ value obtained from the CV for different voltages. Figure 8d shows CV curves recorded at various scan rates from 10 mV/s to 500 mV/s at 2 V. As the scan rate increases, a clear decrease in specific capacitance is observed (inset of Figure 8d), attributed to kinetic limitations. On the other hand, at higher scan rates, ions do not have sufficient time to penetrate into the porous structure of the electrodes. Thus, a limited ion diffusion results in a low *C*_s_ value. At lower scan rates, ions have sufficient time to penetrate deep into the porous electrode structure, enabling full utilization of the active surface area.^50^

The mechanism of charge storage is likely due to (i) redox reaction and (ii) electric double layer formation of the electrode–electrolyte interface. Thus, the diffusive and capacitive contributions were evaluated by Dunn’s method. Figure S2 depicts the capacitive and diffusive contribution and it is quite evident that the SSC is predominantly EDLC type. About 82–88% of capacitance arises due to the formation of the electric double layer.

The galvanostatic charge–discharge (GCD) cycles were obtained at different cutoff voltages to evaluate the operating voltage limit, as shown in Figure 9a. The IR drop is quite low for lower potential values. Evidently, beyond ∼2 V, the IR drop is quite significant. Coulombic efficiency (η) was obtained from $(\frac{\mathrm{discharge}\;\mathrm{time}}{\mathrm{charge}\;\mathrm{time}}\times 100)$ . Figure 9b illustrates the variation of specific capacitance (*C*_s_) and Coulombic efficiency (η) with increasing operating potential. At lower potentials, possibly the sluggish ionic mobility results in reduced *C*_s_ values. Although *C*_s_ increase monotonically with voltage, η exhibits a gradual drop beyond ∼2.0 V operating potentials. Notably, deviations from the ideal triangular shape in galvanostatic charge–discharge (GCD) curves appear beyond 2.0 V, indicating the onset of nonideal behavior or side reactions. Therefore, 2.0 V is identified as the optimum working potential for subsequent electrochemical characterization. At this potential and a current density of 1 mA, a high specific capacitance of ∼ 325 F/g is achieved.

**Figure 9:**
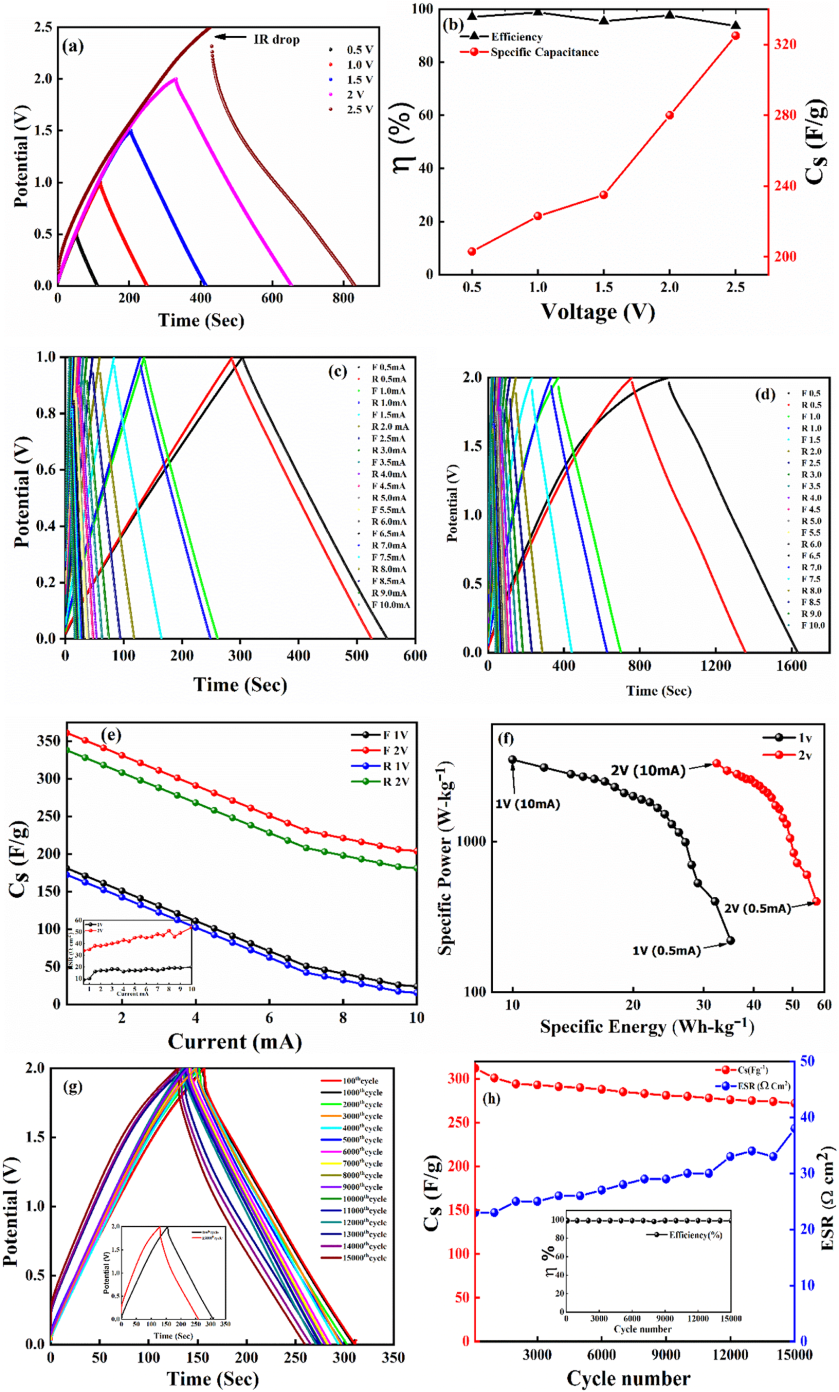
(a) Operating potential optimization of SSC from Galvanostatic charge–discharge (GCD), (b) specific capacitance (*C*_s_) and efficiency (η) from GCD at different operating potential, GCD cycles at different current density value at (c) 1 V, (d) 2 V, (e) capacitance retention at forward and reverse sweep; Inset: ESR at different discharging current for 1 and 2 V (f) Ragone plot at 1 and 2 V, (g) GCD long cycling up to 15000 cycles, (h) specific capacitance and ESR at different cycles; Inset: Coulombic efficiency with cycle number.

Further, the galvanostatic charge–discharge (GCD) cycles were also used to evaluate various performance parameters. The total device capacitance $C=\frac{I\Delta t}{\Delta V}$ (in F) is calculated using discharge current (*I* in A), discharge time (Δ*t* in seconds), and voltage window of the discharge cycle (Δ*V* in volts). The specific capacitance (F/g) per electrode has been calculated as $C_{s}=\frac{2C}{m}$ , where m is the average FD-CA mass on a single electrode. Moreover, the specific energy $\left(E=\frac{1}{2}.\frac{C{\left(\Delta V\right)}^{2}}{3\cdot 6{\;m}_{total}}\right)$ and specific power ( $P=\frac{3600\cdot E_{s}}{\Delta t}$ ) were obtained for the full device in the units of Wh/kg and W/kg, respectively. Furthermore, the equivalent series resistance ( $ESR=\frac{\Delta V_{IR}}{2I}$ ) was calculated using the initial voltage drop during the discharge cycle.

Figure 9c,d display the galvanostatic charge–discharge (GCD) profiles recorded at 40 °C for current densities ranging from 0.5 to 10 mA at 1 and 2 V, respectively. To evaluate the current-dependent stability and capacitance retention of the solid-state supercapacitor (SSC), GCD tests were performed sequentially in both forward (low to high current density) and reverse order. The specific capacitance values extracted from these cycles Figure 9e reveal negligible variation between forward and reverse sweeps, indicating excellent retention and stability across the tested current range. The Inset of Figure 9e shows that the ESR of the SSC does not affect too much even after the discharging current increased by 10 mA, which shows that the SSC is stable up to a wide range of discharging current. Figure 9f presents the Ragone plots for 1 and 2 V operations. At 0.5 mA, the SSC delivers a specific energy of ∼35 Wh/kg at 1 V and ∼57 Wh/kg at 2 V. At 10 mA, the specific power reaches ∼3545 W/kg for 1 V and ∼3500 W/kg for 2 V. These results demonstrate that the SSC maintains high energy and power densities over a broad range of current densities, highlighting its strong potential for practical energy storage applications.

The SSC device was operated at an optimized voltage of 2 V and a current of 2 mA for long-term cycling, as predominant electric double-layer capacitance (EDLC) behavior is observed below 2.5 V. Figure 9g presents the galvanostatic charge–discharge (GCD) curves of the SSC, revealing stable charge–discharge cycles even after 15,000 cycles. The specific capacitance (*C*_s_) and equivalent series resistance (ESR) as functions of cycle number are shown in Figure 9h. The SSC exhibits a high initial specific capacitance of approximately 312 F g^–1^, which gradually decreases to ∼272 F g^–1^ after 15,000 cycles, corresponding to a capacitance retention of about 87%. The Coulombic efficiency remains consistently around 99% throughout the cycling process. Additionally, the ESR remains relatively stable, ranging between 23 Ω cm and 38 Ω cm^2^ during the entire 15,000-cycle test. These results indicate excellent electrochemical stability, with a stable electrode–electrolyte interface and no significant degradation observed over extended cycling.

Table 2 presents a variety of supercapacitors fabricated using carbon aerogel (CA) and activated carbon (AC). These results suggest that the CA is quite suitable for assembling solid-state supercapacitors. In fact, the combination of Al-LLTO-IL electrolyte with CA electrodes exhibits much superior performance than that of liquid based supercapacitor as per the earlier reports.

**Table 2:** Performance Metrics of Supercapacitors Assembled with A Variety of Electrolytes and AC/CA or Other Activated Electrode Materials

s. no.	electrolyte	electrode	findings	ref
1.	30 wt % KOH	pitch-based carbon aerogel (P-CA)	187.2 F g^–1^ at 5 mA cm^–2^	^51^
2.	6 M KOH	activated carbon aerogel containing graphene (ACAG)	300 F g^–1^ at 1A g^–1^	^52^
3.	6 M KOH	carbon aerogel (CA)	110.06 F g^–1^	^53^
4.	30 wt % KOH	activated carbon aerogel (ACA)	245 F g^–1^	^54^
5.	1 M Et_4_NBF_4_–AN solution	CO_2_ activated carbon aerogel	152 F g^–1^, 97% Coulombic efficiency after 10,000 cycles	^55^
6.	1 M Na_2_SO_4_ Aqueous electrolyte	LaFeO_3_	241.3 F g^–1^, 92% capacity retention after 5000 cycles	^56^
7.	3 M KOH solution	LaNiO_3_ and AC	260.45 F g^–1^, 80.78% capacity retention after 10,000 cycles	^57^
8	LATP-13 EMIM BF_4_	AC	181 F g^–1^ and 61% capacitance retention after 13,000 GCD cycles	^42^
9	LLTO-6 EMIM BF_4_	AC	312 F g^–1^ and 60% capacitance retention after 10,000 GCD cycles	^22^
10.	Al-LLTO-IL	FD-CA	360 F g^–1^ and 87% capacitance retention after 15,000 GCD cycles	present work

To summarize the above discussion in three points:

(i) A novel solid-state electrolyte composed of Al^3+^-doped LLTO and ∼6 wt % EMIM BF_4_ was synthesized. Structural stability of the tetragonal perovskite phase was confirmed by Rietveld-refined XRD, XPS, and Raman analyses, indicating successful Al^3+^ incorporation without secondary phases. The incorporation of Al^3+^ further enhances the overall ionic conductivity of the perovskite, which is corroborated by a decrease in activation energy upon doping Al^3+^ into the LLTO matrix. The presence of IL, in fact, couples the grain–grain and the electrode–electrolyte interface. Thus, the contact at the interface is stable without any “butter-layer” to establish the electrode–electrolyte interface. The results suggest that the mesopores in the FD-CA electrodes are effectively utilized by the Li^+^ ions as well as relatively bigger sized IL ions.
(ii) Freeze-dried carbon aerogel (FD-CA) was synthesized and coated on copper foils, was used as the electrode material, offering a high mesoporous surface area conducive to electric double-layer formation. This morphology enabled efficient ion-accessible pathways, enhancing charge storage capacity and rate performance.
(iii) The assembled symmetric solid-state ceramic supercapacitors (SSCs), fabricated via hot-roll lamination, delivered a high specific capacitance (∼370 F g^–1^ at 1 mA/2 V), long-term cycling stability (∼87% retention over 15,000 cycles), and high Coulombic efficiency (∼99%), underscoring their potential for stable and scalable energy storage systems.
(iv) In comparison to previous ionic liquid-based ceramic supercapacitors^58^ and other solid-state gel-based supercapacitors,^53,55–57^ the IL-LALTO combined with FD-CA electrode-based supercapacitors exhibit superior performance and cycling stability.^59–61^

## 4 Conclusions

We have demonstrated the first successful implementation of freeze-dried carbon aerogel (FD-CA) as an electrode material in a solid-state supercapacitor (SSC) configuration employing Al^3+^-doped Li_0.36_La_0.56_Ti_0.995_Al_0.005_O_3_ (Al-LLTO) perovskite as the solid electrolyte. The FD-CA, synthesized via a controlled sol–gel and freeze-drying route, exhibited exceptionally high surface area (∼1600 m^2^·g^–1^), hierarchical porosity, and abundant electrochemically active sites, contributing significantly to the superior electrochemical characteristics of the SSC.

Substitution of Al^3+^ into the LLTO lattice was systematically verified through Rietveld refinement of XRD patterns, XPS, and Raman spectroscopy, all of which confirmed successful substitution at the Ti^4+^ sites without disrupting the perovskite framework. The incorporation of 6 wt % EMIM BF_4_ ionic liquid further enhanced the room-temperature ionic conductivity to the order of 10^–3^ Ω^–1^ cm^–1^, while maintaining the structural integrity of Al-LLTO, as corroborated by XRD.

Finally, the optimized solid-state device demonstrated remarkable long-term electrochemical stability, retaining 87% of its initial capacitance over 15,000 galvanostatic charge–discharge cycles at 2 V and 2 mA, with a high Coulombic efficiency of 99%. This outstanding cycling durability, in conjunction with the synergistic interface between FD-CA and the Al-LLTO-IL (6 wt %) composite electrolyte, underscores the promise of this materials platform for next-generation high-performance and durable all-solid-state supercapacitor.

## Supplementary Material



## Data Availability

All data included in this work are available upon request by contact with the corresponding author.

## Notes

A detailed BET surface area analysis is provided to evaluate the microporous and mesoporous surface areas of carbon aerogel. For a comprehensive understanding of the charge storage process, Dunn’s method analysis is presented to distinguish between the capacitive and diffusive contributions. An original image for EDS mapping (Figure 3d) is included (PDF)
