# Supplementary material for: Solid-State Supercapacitors with Enhanced Performance Using Al3+-Doped Li+ Ion Perovskite Electrolyte Integrated with Carbon Aerogel Electrode
Source: ACS Omega. 2025 Aug 21;10(34):39132–47. doi: 10.1021/acsomega.5c05602 (PMC12409526; doi:10.1021/acsomega.5c05602)
Supplement: Supplementary file 1 [file ao5c05602_si_001.pdf]

Solid-state supercapacitors with enhanced performance using Al^{3+}-doped Li^{+} ion perovskite electrolyte integrated with carbon aerogel electrode

Bhargab Sharma[a], Hardeep[a], Kamaldeep Bisht[a], Ashish Singh[b], Rashmi Singh[b] and Anshuman Dalvi[a*]

[a] Department of Physics, Birla Institute of Technology and Science, Pilani, Pilani Campus, Vidya Vihar, Pilani, Rajasthan 333031, India

[b] Photonic Materials Technology Section, Raja Ramanna Centre for Advanced Technology, Indore 452013, India

*Corresponding author email: adalvi@pilani.bits-pilani.ac.in

Supplementary Material

1. BET surface area analysis

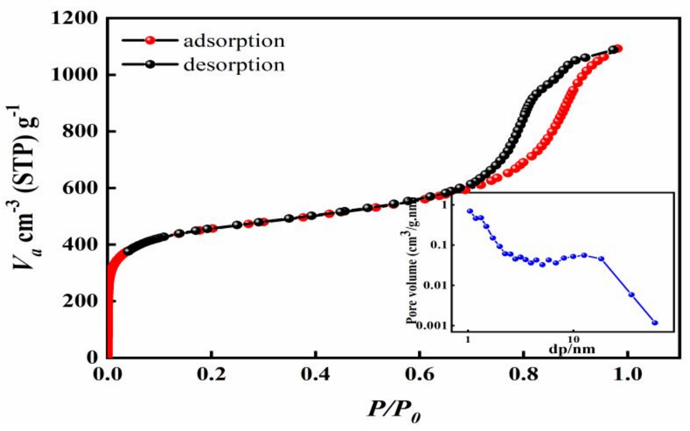

Fig. S1: Nitrogen adsorption-desorption isotherm of FD-CA, inset: pore size distribution.

Fig.S1 illustrates the nitrogen adsorption–desorption isotherm of the freeze-dried carbon aerogel (FD-CA), measured at liquid nitrogen temperature. The isotherm displays a distinct hysteresis loop, characteristic of a Type IV isotherm indicative of mesoporous materials[35]. This hysteresis loop arises from capillary condensation occurring within the mesopores where nitrogen is adsorbed and later desorbed at different relative pressures. Specifically, nitrogen desorbs at a lower relative pressure than it adsorbs, forming the hysteresis loop. The prominent hysteresis observed in FD-CA suggests a significant presence of relatively large mesopores. The onset of capillary condensation at a relative pressure (P/P_0) of ~0.60 further confirms the

dominance of large mesoporous structures. Using the Brunauer–Emmett–Teller (BET) model, the specific surface area of the FD-CA was calculated to be around 1600 m^2/g, reflecting its highly porous and accessible structure. Additionally, the pore size distribution, derived from the desorption branch of the isotherm using the Barrett–Joyner–Halenda (BJH) method and shown in the inset of **Fig. S1** on a logarithmic scale to display the range and distribution of pore sizes more clearly. The distribution curve demonstrates an incremental rise in pore volume with increasing pore diameter, with a majority of the pores falling within the 10–50 nm range. A broad peak in this region readily indicates a wide pore size distribution, reflecting a meso- to microporous structure of FD-CA. From the BJH plot, the mesoporous surface area is ~ 502 m^2g^{-1}. The % of the mesoporous area is around 32%, which suggests that the FD-CA is highly mesoporous in nature. Such a large surface area coupled with well-developed meso to micropore endows the FD-CA with abundant active sites, likely to facilitate efficient electrolyte percolation and rapid ion diffusion throughout the aerogel network.

2. Dunn's Method

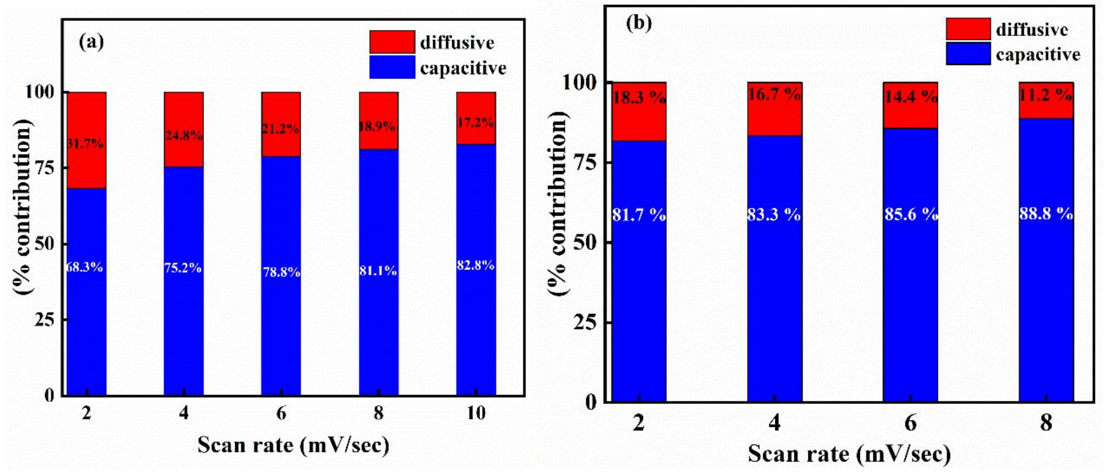

Fig. S2: Capacitive and diffusive contribution for **(a)** 1V **(b)** 2V

The mechanism of capacitor formation is also assessed by evaluating separate contributions of pseudo and electric double layer behavior of the capacitance. This is obtained from CV analysis using Dunn's method[65]. There are various reports on how the current relates to potential scan rate in a typical CV[66]. In a broader sense, the relationship between peak current and scan rate is given by:

$$i_p = av^b \quad \text{(i)}$$

where a and b are constants, whose values decide the nature of the process of charge storage. When the current is controlled by capacitive processes, the b value approaches unity, and when it is controlled by diffusion-controlled processes, it reaches to a value of 0.5[56]. For more complicated systems that involve various charge storage processes, the peak current is a combination of the currents due to capacitive (electrical double layer formation at interface) and diffusion-controlled processes when the ion diffuses into the electrode for a charge transfer as per the following relation:

$$i_p = k_1 v + k_2 v^{1/2} \quad \text{(ii)}$$

where k_1 and k_2 are the constants such that in $i(v)/\, v^{1/2}$ vs v plot gives a straight line with k_1 as slope and k_2 as an intercept. Due to factors including non-porosity of the surface electrode and varied kinetic behaviour at different scan rates, this relationship is not necessarily linear in the required scan rate range[67].

The contribution of pseudo and EDLC to charge storage in supercapacitors is assessed and shown in Fig. S2. It is essential to perform cyclic voltammetry scans at low rates (e.g. 1-10 mV/s) in order to get meaningful information. It is clear that the device is predominantly EDLC type.

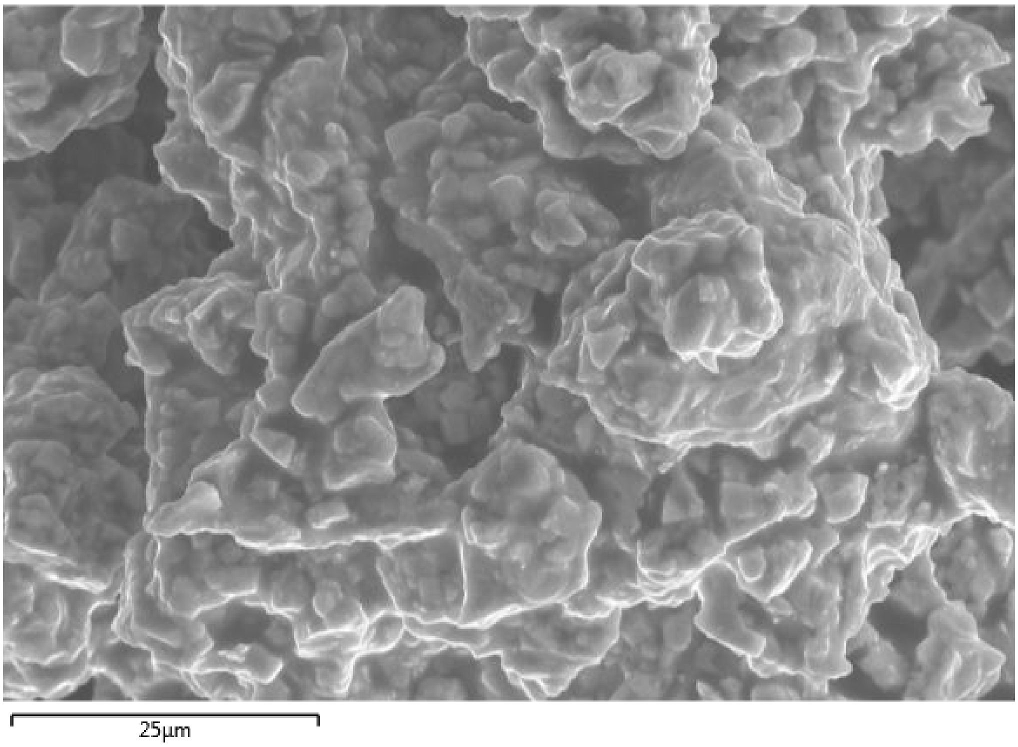

Fig. S3: Original EDS mapping image
